# Patients Have a 15% Redislocation Rate After Arthroscopic Bankart Repair With a Knotless Technique

**DOI:** 10.1016/j.asmr.2023.100864

**Published:** 2024-01-14

**Authors:** Emma Abrahams Nattfogel, Mats C. Ranebo

**Affiliations:** a Department of Orthopedics, Kalmar County Hospital, Kalmar, Sweden; b Department of Biomedical and Clinical Sciences (BKV), Division of Surgery, Orthopedics and Oncology (KOO), Medical Faculty, Linköping University, Linköping, Sweden

## Abstract

**Purpose:**

To evaluate the redislocation rate after arthroscopic Bankart repair (ABR) with a standardized knotless anchor technique in a consecutive series of patients with anterior glenohumeral instability.

**Methods:**

Inclusion criteria were patients who underwent ABR by a single surgeon between January 2008 and December 2018 with a minimum follow up of 2 years. We collected data through phone interviews, Western Ontario Shoulder Instability Index, and review of patient records. The primary outcome was redislocation and secondary outcomes were recurrent subluxations, reoperation, postoperative complications, patient satisfaction, and functional outcomes. We also performed survival analysis and risk factor analysis.

**Results:**

Of 88 patients (91 shoulders) who underwent ABR during the inclusion period, 70 patients (73 shoulders) were included (follow-up rate 80%). The mean follow-up was 7.5 years (range 2-12 years). Redislocation occurred in 15% (95% confidence interval [CI] 7.8%-25.4%) of patients at a mean of 41 months after surgery (range 6-115 months). The reoperation rate for recurrent redislocation was 4.1%. Overall, 90.4% reported being currently satisfied with their shoulder and the mean Western Ontario Shoulder Instability Index score at follow-up was 73.8% (range 8.3%-99.9%). Patients with redislocation were younger at primary operation than patients with no redislocation (mean 21 years vs 28 years; *P* = .023) and adjusted hazard ratio for age was 0.86 (95% CI 0.74-0.99; *P* = .033). It was more common to have less than 3 anchors in patients with redislocation (*P* = .024), but adjusted hazard ratio was 4.42 (95% CI 0.93-21.02; *P* = .061).

**Conclusions:**

The redislocation rate after ABR with a standardized knotless anchor technique in a consecutive series of patients with anterior glenohumeral instability was found to be 15% after a minimum 2-year follow-up (mean 7.5).

**Level of Evidence:**

Level IV, therapeutic case-series.

Arthroscopic Bankart repair (ABR) has become the main surgical treatment option for recurrent anterior glenohumeral dislocations, while the previously more common open Bankart repair is decreasing in use.^1^ Advantages of the arthroscopic technique include low invasiveness, low complication rate,^2^ preservation of the subscapularis tendon, and ability to address concomitant injuries.^3^ Concerns have, however, been raised regarding the high rate of postoperative redislocations, varying between 6.3% and up to 30% in studies with mid- to long-term follow-up.^4^, ^5^, ^6^, ^7^ The vast majority of published literature conveys results from dedicated sports medicine centers or large university clinics.^4^^,^^7^, ^8^, ^9^, ^10^, ^11^, ^12^, ^13^, ^14^, ^15^, ^16^, ^17^, ^18^, ^19^, ^20^, ^21^, ^22^, ^23^, ^24^, ^25^, ^26^, ^27^ It is unknown whether these results can be applied to small regional hospitals carrying out this procedure.

A common topic of discussion and controversy for many orthopaedic procedures is the impact of surgical volume on outcome. Studies on shoulder arthroplasty have found fewer postoperative complications^28^^,^^29^ and significant reduction in readmission rate^30^^,^^31^ in high-volume hospitals. Other studies have found increased surgical complications, length of stay, surgical time, and surgical cost in shoulder arthroplasty and rotator cuff repair when performed by a low-volume shoulder surgeon, defined as <5 procedures per year for arthroplasty and <12 procedures per year for rotator cuff repair.^32^ Studies examining the effect of surgical volume on shoulder stabilization surgery are lacking, however, and the definition of what constitutes high- versus low-volume varies. In a study by Scanlon et al.^33^ on the Latarjet procedure, their low 90-day complication rate and revision rate was attributed to their high surgical volume, with more than 100 procedures per year. Conversely, one study found no significant difference in the redislocation and revision rate after open or arthroscopic shoulder stabilization surgery, when comparing low surgeon volume (≤11/year) and high surgeon volume (≥37/year).^34^ Another study looking at the effect of surgical volume on Bankart repair, performed either arthroscopic or open, showed that high facility volume (>250 cases per year) was associated with surgical failure in their multivariable analysis.^35^

The purpose of this study was to evaluate the redislocation rate after ABR with a standardized knotless anchor technique in a consecutive series of patients with anterior glenohumeral instability. We hypothesized that the rate of redislocation would be approximately 16%, comparable with previously published results.

## Methods

This is a retrospective case series including all consecutive patients with anterior glenohumeral instability who underwent an ABR between January 2008 and December 2018 at our institution. The only exclusion criteria for this study were patients declining participation or inability to contact the patient. At the start of the inclusion period, the surgeon (M.C.R.) had 8 years of experience in arthroscopic knee surgery. This case series includes the first cases of arthroscopic shoulder surgery performed independently by the surgeon (M.C.R.) after supervised learning with an external experienced shoulder specialist during a period of approximately a year. The indication for an ABR in the study period was recurrent anterior dislocations after trauma, defined as 2 or more dislocations or recurrent symptomatic subluxations after 1 traumatic dislocation. At our hospital, patients with anterior instability of the shoulder are primarily treated with ABR as a first-line surgical treatment; however, 17 patients, not included in the study, were treated with an open stabilizing procedure as their primary surgical treatment for anterior instability since 2008. Of these patients, 7 patients were treated with the Latarjet procedure due to a combination of risk factors including glenoid bone loss, number of dislocations, contact sports, as well as patient preference. Nine patients were treated with an open Bankart procedure and one patient with the Bristow procedure by another surgeon at our hospital.

### Surgical Technique

All operations were performed as a day case in a beach-chair position in general anesthesia or sedation, with an interscalene nerve block. The standard antibiotic prophylactic regime was initially one dose of cloxacillin 2 g or clindamycin 600 mg in cases of allergy to penicillin. During 2015, the prophylactic antibiotic regime changed to include a dose of benzylpenicillin 3 g. After a diagnostic arthroscopy through a posterior portal, an anterior-inferior mid-glenoid portal was established over the superior border of the subscapularis. An anterosuperior portal was established in the rotator interval in a similar fashion. The Bankart lesion was mobilized from the anterior surface of the glenoid using a periosteal elevator, hereby also creating a roughened and slightly bleeding bone surface. A Sixter instrument (Depuy Synthes, Raynham, MA) was passed through the inferior glenohumeral ligament and the labrum inferiorly at around 5 o'clock. A non-resorbable FiberWire suture (Arthrex, Naples, FL) was formed into a loop and passed with a Kingfisher instrument (Arthrex) through the anterosuperior portal. The FiberWire loop was caught with the Sixter instrument and passed through the labrum to create a cinch stich. A drill hole for the 3.5-mm PushLock anchor (Arthrex) on the anterior rim of the glenoid aimed to move the labrum as superior and lateral as possible. The suture ends were cut flush after anchor insertion. Another 3.5-mm anchor was then inserted inferior to the first anchor. Depending on intraoperative factors such as the size of the glenoid and the tension of the reattached capsulolabral complex, an additional third anchor was inserted. Some degree of capsulorrhaphy and tensioning of the inferior glenohumeral ligament was thereby performed in conjunction with the labral repair.

We then identified any Hill–Sachs lesion. If intraoperative assessment showed engagement with the glenoid rim, the Bankart repair was augmented with a Hill–Sachs remplissage.^36^ Two double-loaded Fastin RC anchors (DePuy Synthes, Raynham, MA) were inserted into the defect through a posterior cannula. The cannula was then backed out of the infraspinatus tendon and a suture from each anchor retrieved to accomplish one mattress suture per anchor, both tied through the cannula with a sliding knot. The stability of the repair was tested with a load-and-shift test. Postoperatively, the shoulder was placed in an immobilizer sling.

### Postoperative Care and Rehabilitation

Physiotherapy was started after 2 to 4 days. Patients were immobilized with a sling for 3 to 4 weeks. Isometric exercises and passive assisted exercises were started at 2 weeks and increased to active exercises at 4 weeks. Patients had a follow-up with the operating surgeon at 6 weeks and with a physiotherapist at regular intervals until a minimum of 3 to 6 months’ postoperatively. Patients were asked to refrain from contact and overhead sports and risk activities for a total of 6 months.

### Outcome Measures

The primary outcome was recurrent dislocation with at least one postoperative dislocation as reported by patients during a telephone interview at follow-up. The secondary outcome measures include recurrent subluxations, reoperation, postoperative complications such as infections, and the need for additional follow-up appointments at the orthopaedic clinic for symptoms such as pain, stiffness, and clicking of the shoulder. Further outcome measures include patient satisfaction and shoulder function as assessed by the Western Ontario Shoulder Instability Index (WOSI), a condition-specific questionnaire designed for use with patients who have shoulder instability.^37^ The final WOSI score is given as a percentage, where a greater percentage indicates a better function.

### Study Protocol

**Fig 1:**
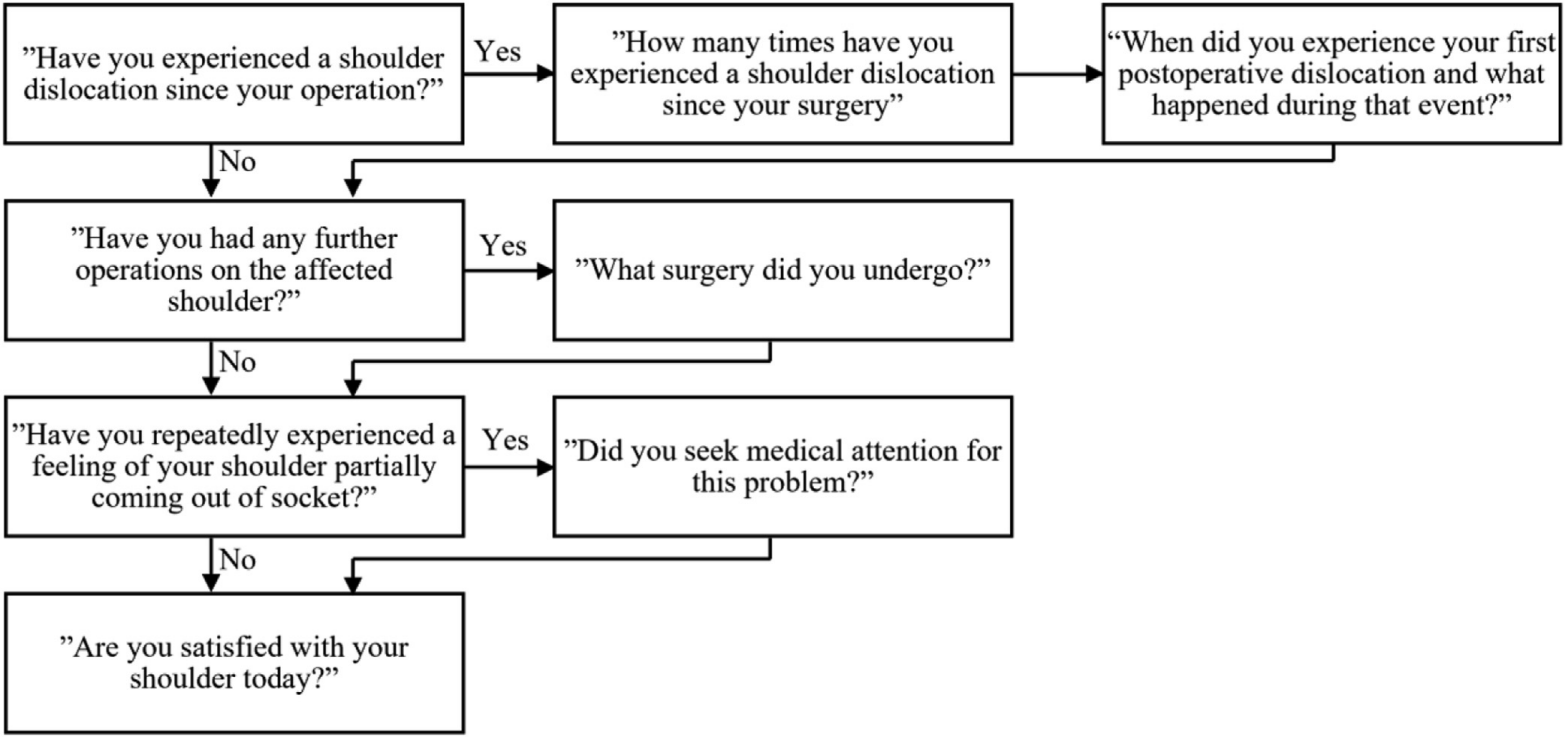
Telephone interview questions.

We obtained Ethics approval from the Swedish Ethical Review Authority (Dnr 2019/05281). We informed all patients of the study via mail, and patients were asked to sign a written consent form and complete a WOSI questionnaire. We then contacted patients via phone and, after verbal consent was given, we asked a series of questions regarding their operated shoulder (Fig 1). We collected information on baseline patient characteristics as well as risk factors, as noted in a recent review,^6^ through systematic examination of the orthopaedic and physiotherapy patient records for the patients who had returned a signed consent form. Extracted data included age, sex, number of preoperative dislocations, comorbidities such as epilepsy, diabetes, psychiatric illness, and smoking. The presence of hyperlaxity, as assessed and documented by an orthopaedic specialist at the preoperative consultation, was noted as well as information regarding participation in sports and at what level.

One author (E.A.N.) assessed preoperative anteroposterior and lateral radiographs of the affected shoulder and noted the presence of osteoarthritis, Hill–Sachs lesion, bony Bankart lesion, and loss of sclerotic line. We calculated the preoperative 10-point instability severity index score (ISIS) for all patients retrospectively.^38^ From the operation notes we collected intraoperative findings including the presence of a Hill–Sachs lesion, a bony Bankart lesion or any cartilage damage. The operative method, including the number of anchors used and whether Hill–Sachs remplissage was performed, was also noted. From the medical charts we also obtained postoperative information on complications, actively searching for information regarding infection, nerve damage, redislocation, reoperation and the presence of additional follow-up appointments requested by the patient as well as whether they were due to pain, stiffness and/or clicking of the shoulder. Pre- and 6-month postoperative WOSI scores, when available, were collected from the physiotherapy records.

### Statistical Analysis

We described patient characteristics with mean and range for continuous data. We checked continuous data for normal distribution with visual inspection of histograms and Q-Q-plots as well as with the Shapiro–Wilks test and, in case of parametric distribution, we used the independent *t*-test to compare groups. If nonparametric distribution was identified, we used the Mann–Whitney *U* test. For group comparison regarding categorical variables, we used the χ^2^ test or Fisher exact test. Preoperative and postoperative WOSI scores were compared with the paired *t*-test. The minimally clinically important difference in a within-subject change for the WOSI score has been found to be 151.9 (7.2%) using a distribution-based method in patients undergoing arthroscopic stabilization surgery.^39^ The primary outcome was the percentage of shoulders with redislocation. We used the Kaplan–Meier method to describe the cumulative probability of having a redislocation and the univariable nonparametric Log-rank test to assess difference in survival time. Univariable Cox regression assessed risk factors for redislocation known from literature,^6^ including sex, age at surgery, age younger than 20 years at surgery, number of anchors (2 or 3 anchors as a binary variable), hyperlaxity (having any degree of hyperlaxity as assessed by including doctor, binary variable), loss of sclerotic line on preoperative frontal projection radiograph and dominant side. We then used multivariable Cox regression to calculate adjusted hazard ratio and corresponding 95% confidence intervals, adjusting for variables with *P* < .15 in univariable analysis. We checked assumptions regarding proportional hazards. We considered a *P* value of <.05 as statistically significant. Analyses were performed with SPSS 24.0 (IBM Corp., Armonk, NY) and Excel (Microsoft, Redmond, WA).

## Results

**Fig 2:**
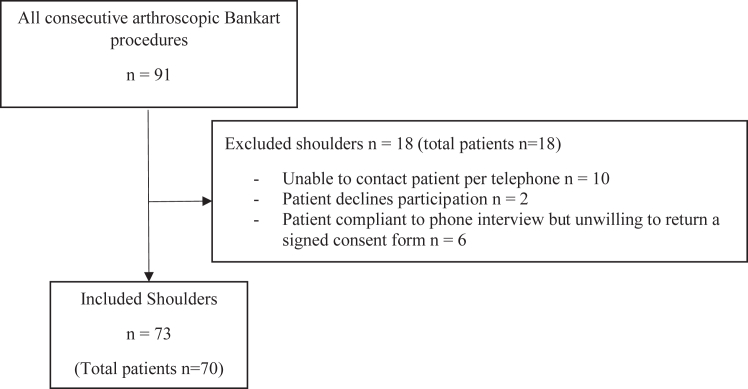
Flow diagram of patient inclusion and follow-up.

Over a period of 11 years, between January 2008 and December 2018, 91 arthroscopic Bankart repairs, with or without Hill–Sachs remplissage, were performed in 88 patients at our county hospital. Of the 88 patients, a total of 70 patients with 73 operated shoulders were included with a follow-up rate of 80% (Fig 2). Only patients with a signed consent form were included in the study. Two patients declined to participate in the phone interview but answered the phone interview questions through a written questionnaire instead.

Patient characteristics are seen in Table 1, including mean age at the time of surgery being 27 years (range 15-58 years). The mean follow-up period was 7.5 years (range 2-12 years). All included patients had either recurrent dislocations (78%) or one dislocation with recurrent frequent symptomatic subluxations (22%) before surgery. Of those with multiple recurrent dislocations, 40% had 2 to 5 dislocations, 18% had 6 to 10 dislocations, and 14% had >10 dislocations. The mean ISIS score was 3 (range 0-8), and 22 patients (23 shoulders) played competitive and/or overhead sports at the time of surgery, including soccer, rugby, basketball, wrestling, ice hockey, handball, floorball, tennis, and swimming.

**Table 1:** Patient Characteristics

Characteristics	All Patients	No Redislocations	Redislocation	*P* Value
Number of shoulders, No. (%)	73	62 (85)	11 (15)	
Follow-up, mean (min–max), y	7 (2-12)	7 (2-12)	9 (3-12)	.067
Age at surgery, mean (min–max), y	27 (15-58)	28 (15-58)	21 (17-30)	**.023**
Age <20 y, No (%)	12 (15)	8 (13)	4 (36)	.075
Male sex, No. (%)	59 (81)	49 (79)	10 (91)	.679
Epilepsy, No. (%)	4 (5)	3 (5)	1 (9)	.488
Current smoker, No. (%)	3 (4)	2 (3)	1 (9)	.392
Dominant arm affected, No. (%)	40 (55)	35 (56)	5 (45)	**.049**
Preoperative dislocations >1, No. (%)	57 (78)	48 (77)	9 (82)	1.000
Shoulder hyperlaxity, No. (%)	38 (52)	30 (48)	8 (73)	.194
Contact∗ or forced overhead sports,† No. (%)	26 (36)	21 (34)	5 (45)	.506
Competitive sports,‡ No. (%)	23 (32)	18 (29)	5 (45)	.306
ISIS score >3, No. (%)	24 (33)	18 (29)	6 (55)	.161
Hill–Sachs lesion preoperative radiographs,§ No. (%)	23 (32)	20 (32)	3 (27)	1.000
Loss of sclerotic line preoperative radiographs,§ No. (%)	11 (15)	10 (16)	1 (9)	1.000
Bony Bankart preoperative radiographs,§ No. (%)	7 (10)	7 (11)	0 (0)	.585
Hill–Sachs lesion intraoperatively, No. (%)	51 (70)	42 (68)	9 (82)	.486
Bony Bankart intraoperatively, No. (%)	14 (19)	13 (21)	1 (9)	.679
Anchors >2, No. (%)	37 (51)	35 (56)	2 (18)	**.024**

NOTE. Values in bold font refers to statistically significant finding.

ISIS, instability severity index score.

∗ Contact sports defined as ice hockey, floorball, football, basketball, rugby, American football, martial arts, and handball.

† Forced overhead sports defined as baseball, handball, swimming, volleyball, water polo and tennis.

‡ Competitive sport defined as any sport in which the patient participates in regular matches as a part of a team regardless of the level of sport.

§ Preoperative radiograph not available in 3 patients.

### Redislocation Rate

Postoperative redislocation occurred in 11 patients, corresponding to 15% (95% confidence interval [CI] 7.8%-25.4%) at a mean of 41 months after surgery (range 6-115 months). Most redislocations (64%) resulted from a traumatic injury. Five patients (45%) had a redislocation within the first 2 years of surgery (Table 2), and only one of them resulted from a distinct trauma. As seen in Table 1, patients in the redislocation group were significantly younger at primary operation than in the group with no redislocation (mean 21 years vs 28 years; *P* = .023). Three anchors, instead of 2, were used in only 2 patients (18%) with redislocation and in 35 patients (56%) without redislocation (*P* = .024). There was however a tendency in more recent years toward the use of 3 anchors, with 6 of 7 patients (86%) being operated with 3 anchors in the last year of the study period. Six patients (55%) with recurrence had an ISIS score greater than 3, compared with 18 (29%) without recurrence (*P* = .616).

**Table 2:** Patients With Postoperative Redislocation

Age	Sex	Number of Dislocations Preoperatively	ISIS Score	Hyperlaxity∗	No. Anchors	FU, y	Time to First Postoperative Dislocation, mo.	Trauma First Postoperative Dislocation	Mechanism of First Postoperative Dislocation	Radiograph- Verified Dislocation	Reop Latarjet†	WOSI at FU, %
22	M	6-10	3	Yes	2	11.8	12	No	Dislocated during sleep	No	No	70.8
30	M	1	1	Yes	2	11.4	80	Yes	Dog pulled hard on lead	No	No	9.5
18	M	6-10	3	Yes	2	11.4	14	No	Bent down to remove a stone under a car	No	No	61.4
20	F	2-5	3	Yes	2	10.8	94	Yes	Bicycle accident	No	No	62.5
22	M	2-5	4	Yes	2	10.3	43	Yes	Climbing accident	No	No	79.6
23	M	1	5	Yes	2	9.0	6	No	Woke up and reached arm over head	No	Yes	36.5
17	M	2-5	5	No	2	8.9	25	Yes	Trauma while playing ice hockey	No	Yes	63.0
19	M	2-5	2	No	2	8.8	115	No	Pushed off bed with arm	Yes	No	69.5
18	M	2-5	6	Yes	3	5.0	27	Yes	Slipped and fell outside	No	No	22.7
20	M	>10	5	No	3	4.4	21	Yes	Fell while playing soccer	No	Yes	53.1
24	M	>10	4	Yes	2	3.3	11	Yes	Fell while playing soccer	No	No	36.2

F, female; FU, follow-up; ISIS, instability severity index score; M, male; Reop, reoperated.

∗ Any degree of hyperlaxity noted during clinical examination preoperatively.

† Reoperated with the Latarjet procedure at time of follow-up.

Eleven further patients (15%) reported some occasional subluxation or sense of instability when asked at follow-up, but no one had contacted the clinic for this problem and 90.9% were satisfied with their shoulder. Another 8 patients (11%) contacted the clinic for an extra postoperative appointment due to pain (2 patients), stiffness (3 patients), or clicking of the joint (1 patient). One of the patients, 38 years old at the primary operation, who contacted the clinic complaining of pain, was subsequently diagnosed with incipient osteoarthritis at 7 years’ postoperatively.

Reoperation rate for recurrent redislocation was 4.1%, and these 3 patients underwent an open Latarjet procedure. One patient, with an age of 49 years at the primary operation, had an additional follow-up appointment due to both pain and stiffness and was subsequently diagnosed with post-traumatic osteoarthritis and underwent a further operation with a shoulder prosthesis at another institution 2 years after the ABR. Another patient, with an age of 26 years at the primary operation, was subsequently followed up at another institution and underwent arthroscopic surgery with tenotomy of the long head of biceps as well as shaving and synovectomy 9 years postoperatively. He was also diagnosed with grade 2 osteoarthritis.

### Intraoperative Findings

Intraoperatively, 51 patients (70%) were found to have a Hill–Sachs lesion (Table 1). Three patients (4%) were found to have a lesion large enough to warrant further treatment with a remplissage procedure. In 14 patients (19%), a bony Bankart injury was identified. Some degree of damage to the cartilage of either the glenoid or the humeral joint surface was found in 21 patients (29%).

### Postoperative Complications

There were no postoperative infections. One patient had a local skin reaction due to extravasation of an intravenous propofol injection. The same patient received 2 extra doses of cloxacillin intravenously in the direct postoperative period. Two patients (6%) had transient greater auricular nerve neuropraxia postoperatively secondary to compression by the headrest in the beach-chair position.

### Functional Outcomes and Patient Satisfaction

Overall, 90.4% reported being currently satisfied with their shoulder during the phone interview. The mean WOSI score at follow-up, was 73.8% (range 8.3%-99.9%) and 78.8% when excluding patients with reoperation and/or redislocation. In 23 patients with a documented preoperative WOSI-score, the mean preoperative WOSI score was 51% (range 27%-82%) and the postoperative WOSI was 78% (range 19%-99%) at a mean follow-up of 5.6 years (*P* < .001). Of these 23 patients, 87% achieved minimally clinically important difference at follow-up. In patients with redislocation the mean WOSI score was 51.3% at a mean follow-up of 9 years. In patients who had reported subluxations or sense of instability the mean WOSI score was 57.1%.

### Survival Analysis and Risk Factor Analysis

**Fig 3:**
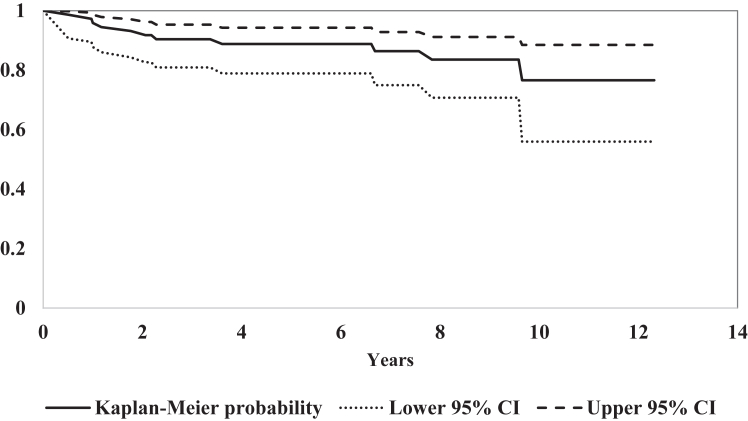
Cumulative probability of being free from redislocation after arthroscopic Bankart repair.

**Fig 4:**
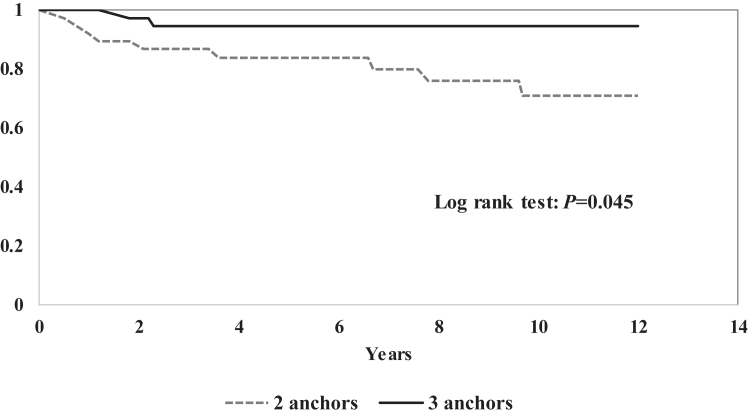
Cumulative probability of being free from redislocation after arthroscopic Bankart repair. Comparison with 2 versus 3 anchors. (CI, confidence interval.)

The cumulative probability of being free from dislocation after ABR is seen in Figure 3. Univariable analysis using the Kaplan–Meier method (Fig 4), with the log rank (Mantel-Cox) test showed that shoulders with 2 anchors had a lower rate of redislocation free survival over time (*P* = .045). In univariable Cox regression, however, the hazard ratio (HR) for this variable was nonsignificant (HR 4.27, 95% CI 0.92-19.95; *P* = .065). In a multivariable Cox regression analysis, only age was a significant factor with a HR of 0.86 (95% CI 0.74-0.99; *P* = .033), adjusting for number of anchors (HR 4.42; 95% CI 0.93-21.02; *P* = .061) and hyperlaxity (HR 3.36; 95% CI 0.81-13.92; *P* = .095).

## Discussion

The results showed a recurrent dislocation rate of 15% and reoperation rate of 4%, after a minimum 2-year follow-up (mean 7.5 years) after ABR. Furthermore, there were no postoperative infections. An overview of studies with mid- to long-term results after ABR can be found in Table 3.^4^^,^^7^, ^8^, ^9^, ^10^, ^11^, ^12^, ^13^, ^14^, ^15^, ^16^, ^17^, ^18^, ^19^, ^20^, ^21^, ^22^, ^23^, ^24^, ^25^, ^26^, ^27^ The majority of these studies were undertaken at dedicated sports medicine clinics and university hospitals. Redislocation rate after ABR varies considerably, as do inclusion criteria and patient selection. The mean redislocation rate in these studies are 16.6% (6.3%-30%), at a mean follow-up of 9.5 years. We found a similar redislocation rate at a regional hospital with a low but comparable operative volume.

**Table 3:** Overview of Studies With Mid- to Long-Term Results After Arthroscopic Bankart Repair

Study	Sample Size, n	Op/Inclusion Period, n	Inclusion Period, y	FU Mean, y	FU Range, y	FU Rate, %	Clinic	Op Volume, cases/year	Redislocation Rate, %
Rhee et al., 2006^8^∗	16	nr	7	6	3-11	nr	Uni	nr	18.8
Kim et al.,^4^ 2009	32	nr	10	6	5-7	nr	Uni	nr	6.3
Owens et al., 2009^9^†	40	49	6.5	11.7	9.1-13.9	81.6	Uni	8	14.3
Castagna et al., 2010^10^	31	43	2	10.9	10-14	71.4	Uni	22	19.3
Kavaja et al., 2012^11^	83	187	5	13	11-15	82.2	Uni	37	22.9
Privitera et al., 2012^12^	20	32	7	13.5	10.8-17.5	63	Uni	5	25
Zaffagnini et al., 2012^13^	49	110	9	13.7	11.5-15.9	74.8	Sm	12	12.2
Boughebri et al., 2014^14^	45	nr	5	6,6	5-10	80	Sm	nr	8.9
Plath et al., 2015^15^	100	165	4	13	10-17	60.6	Uni	41	21
Chapus et al., 2015^16^	18	21	1.5	9.7	8-11	85.7	Uni	14	25
Aboalata et al., 2016^17^	143	180	5	13.3	nr	79.4	Sm	36	18.2
Zimmermann et al., 2016^18^	271	299	10	12.2	nr	90.6	Uni	30	13.3
Gasparini et al., 2016^19^	143	230	12	6.8	2-14	81.7	Uni	19	13
Yang et al., 2018^20^	160	180	13.5	6.4	2-12.5	88.9	Uni	13	13.7
Loppini et al., 2019^21^	670	850	7	8.4	5.2-13.1	78,8	Uni	121	17
Thomazeau et al., 2019^22^	105	328	1	9	nr	84	Sm	nr	11.4
Kanatli et al., 2019^23^	87	169	5.5	6.8	4-11.6	90.6	Uni	31	8
Van Gastel et al., 2019^24^	71	104	7	13.1	nr	68.3	Tech	15	9.9
Vermeulen et al., 2019^25^	147	220	9	6.3	3-12	66.8	Sec	24	14.3
Yapp et al., 2020^26^‡	33	nr	3.5	14.2	12-16	74	Uni	nr	12.1
Panzram et al., 2020^27^	100	nr	11	8	3-14	nr	Uni	nr	22
Yian et al., 2020^7^§	337	540	6	6.2	3.4-9.3	62.4	nr	nr	30
Mean	119	203	6.3	9.2		76.0		28.5	16.6
Nattfogel et al. (current study)	73	91	11	7	2-12	80.2	Reg	8.3	15.0

FU, Follow-up; nr, not reported in original publication; nr, not reported; Op, operation; Reg, regional hospital; Sec, shoulder and elbow center; Sm, sports medicine clinic; Tech, teaching hospital; Uni, university clinic.

∗ Only collision athletes.

† Only first-time dislocations and young athletes.

‡ Only first-time dislocations and part of a randomized trial.

§ Multicenter study.

A possible reason includes the use of a knotless technique, allowing a superior translation of the labrum and tensioning of anterior band of the inferior glenohumeral ligament. We find this to be a more reproducible technique than inserting a suture anchor first; however, when we examined the published literature, no significant difference in outcome was found between knotless and knotted anchors for labral repair, including Bankart repair.^40^

The definition of what constitutes failure after shoulder stabilization varies between studies. A study by Zimmermann et al.^18^ found a redislocation rate of 13.3% at a mean follow-up of 6 years and defined redislocation as any documented dislocation requiring reduction by a third party or medical professional. A similar definition was used in a study by Van Gastel et al.^24^ Yapp et al.^26^ defined recurrent dislocation as a radiographically confirmed dislocation. Some studies instead report recurrent instability including both dislocations and subluxations as their main outcome and thereby generally reporting higher recurrence rates.^5^^,^^41^^,^^42^ Van der Linde et al.^5^ found a redislocation rate of 35.3% at a mean follow-up of 9 years in their consecutive series of 68 patients older than the age of 18 years while another study looking only at patients younger than the age of 18 years had a recurrence rate of 57.1% at a mean follow-up of 12.2 years.^42^ These apparent differences in definitions must be taken into consideration when comparing results as studies with wider definitions of recurrence tend to report a greater recurrence rate.

Almost one half (45.5%) of recurrent dislocations occurred within 2 years of the primary operation. This finding is similar to other published results.^5^^,^^27^^,^^41^^,^^43^ The mechanism of injury in 3 of 5 patients with redislocations that occurred within the first 2 years were atraumatic in nature (Table 2). Possible reasons for these kinds of failure could be perioperative technical problems, delayed healing of the repair, insufficient rehabilitation, insufficient proprioceptive regain or poor patient selection. The other 2 early dislocations were due to substantial trauma during soccer.

The redislocation risk with fewer anchors seemed to be higher in a univariable analysis but did not reach statistical significance in an adjusted multivariable model, possibly due to small sample size. Results are inconsistent in previous literature regarding this, some early studies have found this to be a significant risk factor^38^^,^^44^ but not in later studies.^5^^,^^6^ Several other risk factors for recurrent dislocation after ABR have been found in a recent meta-analysis using results from 29 studies and 4,578 patients^6^: age, competitive sports, Hill–Sachs lesion, glenoid bone loss, anterior labroligamentous periosteal sleeve avulsion lesion, >1 preoperative dislocation, >6 months’ delay, and ISIS >3 and ISIS >6.

We had a reoperation rate of 4%, which is lower than the 11% to 21% reported in literature.^18^^,^^24^^,^^26^^,^^27^^,^^41^ Reoperation rates should, however, be interpreted with caution, since this is dependent on local routines and complex decision-making among treating surgeons and patients.

The WOSI scores at long-term follow-up in our study (mean 73.8%) were found to be similar to those in published literature. In Flinkkilä et al.,^41^ the mean WOSI core was 80% at 12 years and Vermeulen et al.^25^ found the WOSI score to be 54.8% in patients with recurrence and 81.4% in patients with no recurrence compared to the results in our study with WOSI score of 51.3% (9.5-79.6%) and 78.8% (8.3-99.9%) in respective groups.

Our results show that 15% of patients who did not experience a postoperative dislocation reported some occasional subluxation or sense of instability at a mean follow-up of 7.5 years postoperatively. It is, however, difficult to determine the clinical significance of this finding, considering that 90% of this group of patients were satisfied with their shoulder function at follow-up. These patients could have some minor instability in other directions that is not addressed by the Bankart repair or other symptoms not directly related to the Bankart problem, although it could also represent a subclinical failure of the labral repair.

Patient satisfaction was high at long-term follow-up, with 90.4% of patients reporting to be currently satisfied with their shoulder. Similarly, in a study by Yapp et al.,^26^ 83% of patients considered themselves satisfied or very satisfied with their overall treatment. In a study by Van Gastel et al.,^24^ 39.7% of patients reported being completely satisfied, 45.2% reported being mostly satisfied, and 11% reported being somewhat satisfied. In the study by Vermeulen et al.,^25^ 89% of patients reported being satisfied with their treatment.

Strengths of this study include a relatively long follow-up, with a mean follow-up period of over 7 years, including all consecutive patients who had undergone Bankart repair for anterior instability.

## Limitations

Quantification of glenoid bone loss and Hill–Sachs lesions radiographically was not possible because of the lack of preoperative computed tomography or magnetic resonance imaging scans in our patients. This limits our risk factor analysis and the characterization of the patients. We did not collect information about the date of the first dislocation relative to the date of surgery, a characteristic that could affect the risk of redislocation post-operatively. Because of the retrospective methodology, we would expect a high risk of recall bias when asking this question. Hyperlaxity is not precisely defined and described in detail due to the retrospective nature of the study. A more precise quantification of hyperlaxity with a preoperative Beighton score would have been preferable. We defined competitive sport as any sport in which the patient participates in regular matches as a part of a team regardless of the level of sport. As a result, it is possible that the ISIS score may have been overestimated in some cases. However, similar definitions can be found in studies examining the ISIS scoring system as a predictor of redislocation.^45^^,^^46^

During the later years of the study, some selection bias could have occurred as we started treating some patients with primary Latarjet procedures instead of Bankart repair, thereby possibly excluding some patients with a higher redislocation risk. We were not able to examine patients physically or radiographically at follow up thereby limiting some aspects of results, for example range of motion and development of osteoarthritis.

## Conclusions

The redislocation rate after arthroscopic Bankart repair with a standardized knotless anchor technique in a consecutive series of patients with anterior glenohumeral instability was found to be 15% after a minimum 2-year follow-up (mean 7.5 years).

## Disclosure

All authors (E.A., M.C.R.) declare that they have no known competing financial interests or personal relationships that could have appeared to influence the work reported in this paper. Full ICMJE author disclosure forms are available for this article online, as supplementary material.

## Supplementary Data

ICMJE author disclosure forms
